# Small bowel obstruction complicating colonoscopy: a case report

**DOI:** 10.1186/1752-1947-2-179

**Published:** 2008-05-27

**Authors:** Iain A Hunter, Rupa Sarkar, Andrew M Smith

**Affiliations:** 1 St. James's University Hospital, Beckett Street, Leeds, West Yorkshire LS9 7TF, UK; 2 Scunthorpe General Hospital, Cliff Gardens, Scunthorpe, North Lincolnshire, DN15 7BH, UK

## Abstract

**Introduction:**

This report describes a rare complication of colonoscopy and reviews the literature with regard to other rare causes of acute abdominal presentations following colonoscopy.

**Case presentation:**

After a therapeutic colonoscopy a 60-year-old woman developed an acute abdomen. At laparotomy she was discovered to have small bowel obstruction secondary to incarceration through a congenital band adhesion.

**Conclusion:**

Although there is no practical way in which such rare complications can be predicted, this case report emphasises the wide array of pathologies that can result in acute abdominal symptoms following colonoscopy.

## Introduction

Colonoscopy is a widely used investigative procedure and has a relatively low complication rate. With the advent of the National Health Service Bowel Cancer Screening Programme [1] there will be an increase in the number of procedures being performed in the UK. This will result in an inevitable increase in the number of colonoscopy-related complications requiring acute hospital admission. This report describes a rare complication of colonoscopy and reviews the literature with regard to other rare causes of acute abdominal presentations following colonoscopy.

## Case presentation

A 60-year-old woman presented to an outpatient colorectal clinic with a 2-month history of rectal bleeding. Her past medical history included a colpopexy which had been performed via a Pfannenstiel incision 20 years earlier. On examination a large villous adenoma was palpable within the rectum. She was referred for a diagnostic colonoscopy. The colonoscopy was performed without immediate complication using 25 μg of fentanyl and 2 mg of midazolam. Bowel preparation was performed to good effect with 2 litres of polyethylene glycol solution. The exclusion value of the examination was reported as excellent. Caecal intubation was confirmed by visualisation of the ileocaecal valve. The terminal ileum was not intubated. Two 4 mm sessile polyps were located within the middle third of the rectum and these were removed using hot biopsy. The lower third of the rectum contained a large sessile villous adenoma which occupied 50% of the rectal circumference at this level (7 cm diameter). The lesion was sampled by plain biopsy. Histological analysis revealed the 4 mm polyps to be tubulovillous adenomas and the larger sessile polyp to be a villous adenoma.

**Figure 1:**
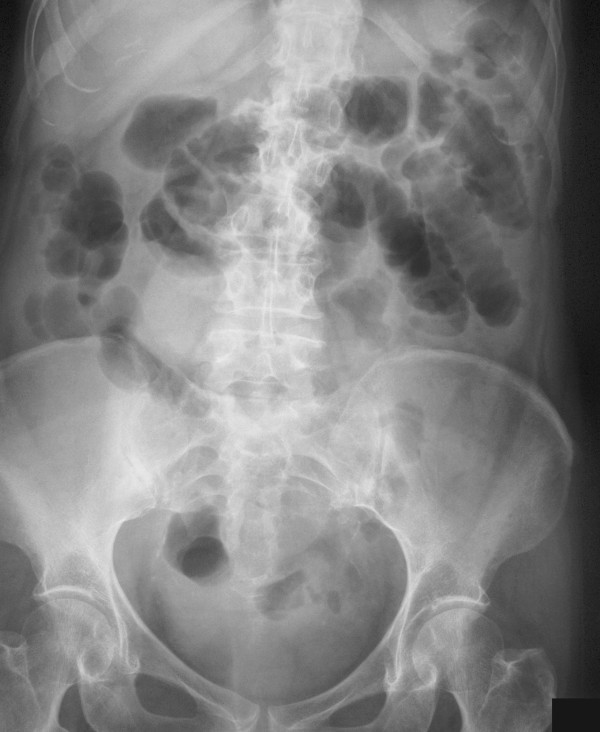
**Plain abdominal radiograph**. This radiograph was captured 16 hours after colonoscopy. Several loops of dilated and oedematous small bowel are visible in the left upper quadrant of the abdomen.

Eight hours after colonoscopy the patient developed gradual onset of abdominal pain associated with nausea and vomiting. She was admitted under the acute surgical service the next day. On examination she was afebrile with a heart rate of 130 beats per minute. Abdominal examination revealed diffuse tenderness with fullness and peritonism in the right iliac fossa. Peripheral blood analysis demonstrated a white cell count of 16.9 × 10^9^/litre. Plain abdominal and chest radiography demonstrated several loops of dilated small bowel in the left upper quadrant but no evidence of free abdominal gas (Figure 1). The patient was assumed to have a post-colonoscopic perforation with a resulting ileus. At laparotomy the mid-ileum was found to be strangulated within a congenital band adhesion. The adhesive band was localised to the right iliac fossa and was well removed from the site of her previous pelvic surgery. The 30 cm herniated intestinal segment was non-viable and was resected. Continuity was restored with a primary end-to-end anastomosis. The patient made an unremarkable recovery and was discharged home 6 days later.

## Discussion

The development of an acute abdomen after colonoscopy is a relatively rare event. Overall rates of colonic perforation are widely reported at being in the region of 0.12% (see [2]). Although colonic perforation is the most common cause of acute abdomen following colonoscopy, several other aetiologies have been reported. A Medline search (Data base: 1950 to 2007; Search term: Colonoscopy; Subheading: Adverse Effects; Limitations: Case Reports) discovered 49 reports of splenic injury, 14 cases of appendicitis, 10 cases of ischaemic colitis, 5 cases of small bowel perforation, 3 cases of cholecystitis, 3 cases of portal pyaemia, 2 cases of small bowel arterial thrombosis, 1 case of pancreatitis and 1 case of a ruptured iliac aneurysm following colonoscopy.

Mechanical bowel obstruction following colonoscopy has been reported by 21 other authors. Half of these have been related to volvulus of the caecum, sigmoid or entire small bowel mesentery. One case of caecocolic intussusception has been reported [3]. Three cases describe the resolution of small bowel ileus or obstruction following conservative treatment. The authors note that each of these episodes occurred in patients who had previously had appendicectomy or colonic resection, suggesting that they are cases of adhesive small bowel obstruction precipitated by colonoscopy [4,5]. This theory is supported by the observation of two cases requiring laparotomy and adhesiolysis of post appendicectomy adhesions [6,7]. The remainder are related to incarceration within external or internal hernias. Inguinal hernias have accounted for three cases whilst one case of large bowel diaphragmatic herniation is reported.

Our case is one of only five in the literature relating to the internal incarceration of small bowel as demonstrated at laparotomy. These include a case of colonoscopy-induced sigmoid mesenteric rupture and subsequent small bowel incarceration through the defect [8], one case related to a postcaecocystopexy band adhesion [9], one case of ileal incarceration in a paracaecal hernia [10] and one case of incarceration in a mesenteric defect [4]. It seems likely that inflation of the colon and small bowel combined with extensive manipulation is responsible for the development of internal incarceration. Such complications may be minimised by a good colonoscopic technique. This should utilise torque steering and the avoidance of extensive insufflation and pushing in order to maintain a straight scope and a short colon.

## Conclusion

Although there is no practical way in which such rare complications can be predicted, this case report emphasises the wide array of pathologies that can result in acute abdominal symptoms following colonoscopy. Such presentations require a high index of suspicion on the clinician's part with a low threshold for urgent investigation and intervention.

## Competing interests

The authors declare that they have no competing interests.

## Consent

Written informed consent was obtained from the patient for publication of this case report and any accompanying images. A copy of the written consent is available for review by the Editor-in-Chief of this journal.

## Authors' contributions

IAH was responsible for drafting the manuscript and obtaining informed consent from the patient, RS contributed to research and review of the relevant literature, AS conceived of the report and edited the draft manuscript.
